# Nonlinear Optics in Two-Dimensional Magnetic Materials: Advancements and Opportunities

**DOI:** 10.3390/nano15010063

**Published:** 2025-01-02

**Authors:** Ziqian Xin, Bingyuan Xue, Wenbo Chang, Xinping Zhang, Jia Shi

**Affiliations:** Institute of Information Photonics Technology, School of Physics and Optoelectronics, Faculty of Science, Beijing University of Technology, Beijing 100124, China; xzq13266910259@emails.bjut.edu.cn (Z.X.); xuebingyuan@emails.bjut.edu.cn (B.X.); wenbo060402@126.com (W.C.); zhangxinping@bjut.edu.cn (X.Z.)

**Keywords:** two-dimensional magnetic materials, SHG, magnetic order

## Abstract

Nonlinear optics, a critical branch of modern optics, presents unique potential in the study of two-dimensional (2D) magnetic materials. These materials, characterized by their ultra-thin geometry, long-range magnetic order, and diverse electronic properties, serve as an exceptional platform for exploring nonlinear optical effects. Under strong light fields, 2D magnetic materials exhibit significant nonlinear optical responses, enabling advancements in novel optoelectronic devices. This paper outlines the principles of nonlinear optics and the magnetic structures of 2D materials, reviews recent progress in nonlinear optical studies, including magnetic structure detection and nonlinear optical imaging, and highlights their role in probing magnetic properties by combining second harmonic generation (SHG) and multispectral integration. Finally, we discuss the prospects and challenges for applying nonlinear optics to 2D magnetic materials, emphasizing their potential in next-generation photonic and spintronic devices.

## 1. Introduction

In the fields of nanophotonics and quantum materials research, 2D materials have garnered significant attention in recent years due to their unique layered structures and quantum confinement effects, which endow them with a host of novel optical properties [1,2,3,4,5,6,7]. Among these, the discovery of 2D magnetic materials, particularly single-layer or few-layer magnetic materials such as CrI_3_ [8] and Fe_3_GeTe_2_ [9], has greatly advanced the intersection of spintronics and photonics [10,11,12]. These materials not only exhibit ultra-thin structures but also display rich intra- and interlayer magnetic coupling effects, which can be significantly modulated by external field, such as electric fields, magnetic fields, or mechanical strain. This tunability positions 2D magnetic materials as promising candidates for the development of multifunctional and innovative photonic devices.

Nonlinear optics, as a fundamental branch of modern optics, holds a prominent position in physics and materials science. In this context, the phenomenon of SHG, a key nonlinear optical effect, has been widely employed in applications such as frequency-doubling lasers, optical sensing, and material characterization [13,14,15,16]. When applied to 2D magnetic materials, SHG provides a powerful tool to study the interaction between light and magnetism, enabling the exploration of physical phenomena such as symmetry breaking and magnetic state transitions [17]. These studies not only provide theoretical insights but also offer technical support for designing efficient and innovative photonic devices. This expands the scope of nonlinear optics research while opening new avenues for integrating 2D materials into photonic technologies.

This review summarizes the magnetic structures of 2D magnetic materials and the principles of SHG. It explores the use of SHG to investigate structural and magnetic properties, including layer-dependent and polarization-resolved studies. Additionally, we discuss the role of SHG in detecting magnetic dynamics and conclude with an outlook on future challenges and opportunities for integrating nonlinear optics with 2D materials.

## 2. Overview of Magnetic Characteristics and the Basic Principles of SHG

### 2.1. The Magnetic Structure of 2D Magnetic Materials

Two-dimensional (2D) magnetic materials hold great promise for applications in optoelectronics, magnetic storage, and spintronic devices [12,18,19,20,21], making them a focal point of contemporary research. Early studies on magnetism in 2D materials primarily introduced magnetic properties through doping, defects, or vacancies [22,23,24,25]. However, these approaches often resulted in weak magnetism that was difficult to control precisely. Consequently, the discovery of intrinsically magnetic 2D materials has become a priority. In 2017, Xiaodong Xu [8] and Xiang Zhang et al. [26] reported on monolayer CrI_3_ with strong intrinsic ferromagnetic order and anisotropy, as well as few-layer Cr_2_Ge_2_Te_6_ stabilized by an external exchange field. These pioneering studies marked the beginning of a new era in the study of monolayer and few-layer 2D magnetic materials, laying the groundwork for the discovery of stable 2D magnetic materials with high transition temperatures.

**Figure 1:**
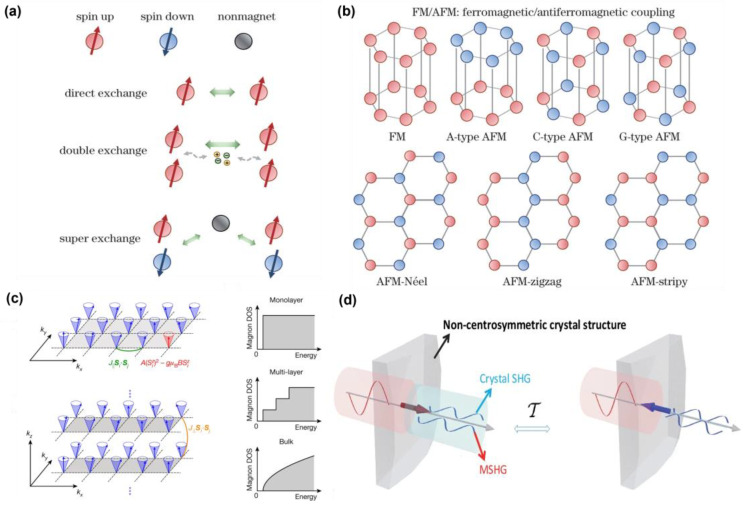
(**a**) Schematic diagram of direct exchange, double exchange, and superexchange between atomic magnetic moments. (**b**) Classification of magnetic configurations of ferromagnetic and antiferromagnetic materials The red circle represents spin up, the blue circle represents spin down, and the gray circle represents nonmagnetic [62]. (**c**) Schematic of spin wave excitation in two-dimensional and three-dimensional systems, with intralayer and interlayer exchange interactions $J_{∥}$ (in green) and $J_{⊥}$ (in orange), respectively. The right side illustrating the spin magnon density of states (DOS) around the low-energy band edges for single-layer, multi-layer, and bulk ferromagnetic materials [26]. (**d**) Schematic diagram of the nonlinear magneto-optical effect [63]. panel (**a**,**b**) adapted with permission from Ref. [62], Copyright 2023, Chinese Journal of Lasers; panel (**c**) adapted with permission from Ref. [26], Copyright 2017,Nature; panel (**d**) adapted with permission from Ref. [63], Copyright 2024, npj computational materials.

These advancements have significantly accelerated the exploration of single-layer and few-layer magnetic materials, opening up new possibilities for their integration into real-world applications. Controllable 2D magnetic materials offer immense potential for the development of high-performance devices across a wide range of technologies, underscoring their importance in both fundamental research and applied science. Two-dimensional materials are mainly divided into four categories: ferromagnetic, ferrimagnetic, paramagnetic, and antiferromagnetic materials. Although the four types of magnetic materials each have their own unique properties, ferromagnetic and antiferromagnetic materials are more critical from the perspective of magnetic storage [27] and spin electronics applications [28].The magnetic moment of magnetic materials generally comes from the spin and orbital angular momentum of the transition metal 3d orbital, and the exchange interaction between transition metal ions plays a key role, which can form a spin arrangement between the ions and drive the long-range order inside the magnetic material, so that the material presents a stable magnetic structure such as ferromagnetic or antiferromagnetic [29]. As shown in Figure 1a, the magnetic moment between two atoms (i and j) typically produces exchange energy, which can be described in terms of the Heisenberg spin Hamiltonian (H), $H=\sum_{i,j}JS_{i}S_{j}$ where S is the spin operator and J is the exchange coefficient [30]. In the magnetic system, the exchange mechanism mainly includes direct exchange, double exchange, and hyperexchange [31]. Direct exchange is caused by the direct overlap of the wave function between the electrons of the transition metal d and is a short-range interaction, in contrast to the hyperexchange interaction that is prevalent in magnetic materials [32,33]. A hyperexchange interaction is an indirect exchange that uses non-magnetic atoms as a medium to extend short-range interactions further [34,35,36]. The double exchange interaction generally exists between magnetic ions in different valence states, and generally exists in metal systems. Neutron scattering (NS), electron spin resonance (ESR), and density functional theory (DFT) each offer unique insights into magnetic interactions, with complementary strengths and limitations. NS directly probes spin excitations and is particularly effective in distinguishing between direct exchange and superexchange interactions, though it requires large-scale facilities and may struggle with weak or low-energy interactions. For example, H. Jacobsen et al. used inelastic NS to investigate spin dynamics in CuO, finding that the high-energy spectra matched the dispersion of an ideal S = 1/2 Heisenberg antiferromagnetic chain, while the low-energy spectra exhibited three-dimensional characteristics, providing insight into the exchange constants of the effective spin Hamiltonian [37]. C.L. Saiz et al. distinguished the contributions of direct exchange and superexchange interactions in CrI_3_ by analyzing temperature-dependent ESR spectra, where changes in linewidth and resonance field allowed them to differentiate the magnetic contributions [38]. However, ESR lacks the precision to quantitatively differentiate between various types of exchange interactions, making it less suitable for detailed analysis compared to NS or DFT. DFT, on the other hand, offers the most detailed and quantitative analysis of exchange interactions. Cheng Gong et al. used first-principles DFT calculations to quantify the contributions of direct exchange and superexchange interactions in 2D ferromagnetic materials like CrI_3_. Their study shows that direct exchange, particularly between nearest-neighbor spins, plays a significant role in ferromagnetism, while superexchange influences interlayer interactions [26]. However, DFT requires substantial computational resources, and the results are highly dependent on the choice of exchange-correlation functionals. In summary, while NS and ESR provide valuable experimental insights into the nature of magnetic interactions, DFT offers a detailed theoretical framework for quantifying these interactions. Each method has its strengths, and combining them can help overcome their individual limitations, leading to a more comprehensive understanding of magnetic ordering in complex systems. As shown in Figure 1b, when J > 0, the two local magnetic moments tend to be arranged in parallel, and the material is ferromagnetic, and when J < 0, the two local magnetic moments tend to be arranged in antiparallel, and the material is antiferromagnetic [39]. The magnetic order of antiferromagnetic materials can be classified according to the intralayer and interlayer coupling and the interlayer coupling can be divided into A-type antiferromagnetic [40,41,42] (intralayer ferromagnetic coupling, interlayer antiferromagnetic coupling), C-type antiferromagnetic [43] (intralayer ferromagnetic coupling, interlayer ferromagnetic coupling), and G-type antiferromagnetic [44] (intralayer and interlayer antiferromagnetic coupling) can be divided into Néel, stripy, and zigzag type antiferromagnetism [45,46,47]. According to the Mermin–Wagner theorem [48], short-range interactions with continuous symmetries in 2D systems cannot sustain long-range order at finite temperatures. However, magnetic anisotropy breaks this rotational symmetry, reducing sensitivity to thermal fluctuations. As shown in Figure 1c, it introduces a gap in the spin-wave energy spectrum, significantly raising the activation temperature of low-energy excitations, thus stabilizing long-range magnetic order [26]. Therefore, magnetic anisotropy is crucial for maintaining magnetic order in 2D systems. It can be enhanced through strategies such as tuning external fields (e.g., pressure, magnetic fields) to modify the material’s bandgap or by designing heterostructures that combine materials with different magnetic anisotropy characteristics and induce additional effects via external fields [49,50]. These approaches are key to improving magnetic anisotropy and the thermal stability of the system.

At present, most spintronic devices use ferromagnetic materials that are easy to understand and control [33,51,52]. However, the parasitic magnetic fields of ferromagnetic materials may interfere with each other and affect device performance. A possible solution is to replace ferromagnetic materials with antiferromagnetic materials. Although antiferromagnetic materials have no macroscopic magnetization, many key phenomena in ferromagnetic systems, such as spin torque [53], giant magnetoresistance (GMR) [54], tunneling anisotropic magnetoresistance (TMR) [55], etc., also exist in antiferromagnetic systems. In addition, antiferromagnetic materials have a richer variety than ferromagnetic materials [56], and because they have zero or near-zero magnetization, they do not produce additional magnetic field interference like ferromagnetic materials. Therefore, antiferromagnetic materials have shown unique advantages in spintronic applications.

However, due to the lack of macroscopic magnetization in the antiferromagnetic state, the material system as a whole does not show a net magnetic moment to the outside, which makes traditional magnetic measurement methods (such as magneto-optical Kerr effect (MOKE) [57], and magnetic circular dichroism (MCD) [58]) unable to effectively detect the magnetic characteristics of antiferromagnetic materials. In addition, antiferromagnetic materials are usually atomically thin, which makes it impossible for them to provide effective magnetic order signals through traditional detection methods such as neutron scattering, vibrating sample magnetometer (VSM), and conducting quantum interference device (SQUID). In response to these challenges, magneto-optical [59,60] SHG as shown in Figure 1d has become a powerful tool for studying the magnetic characteristics of two-dimensional magnetic materials [17,61]. Compared with traditional methods, magneto-optical SHG spectrum is highly sensitive to the structural symmetry of the material and is not limited by quantum efficiency. Therefore, it can effectively detect the magnetic properties of magnetic materials, especially in low-dimensional materials.

### 2.2. Basic Principles of SHG

In 1961, Franken et al. [64] discovered that the ruby laser (694.3 nm) produced a new spectral line with a wavelength of 347.15 nm after passing through the quartz crystal, and the frequency of the newly generated light was twice the frequency of the incident light, that is, the frequency doubling phenomenon, also known as the optical SHG, which was the beginning of nonlinear optics.

Before introducing the SHG, we must first explain the optical sum frequency [59]: two laser beams with incident frequencies of $\omega_{1}$, $\omega_{2}$ are incident on a nonlinear crystal. The nonlinear optical effect caused by the two laser beams generates a nonlinear polarization intensity with a frequency of $\omega_{3}=\omega_{1}+\omega_{2}$. For the second harmonic, when the incident frequencies of the two laser beams are $\omega_{1}=\omega_{2}=\omega$, that is, $\omega_{3}=2\omega$, the second harmonic will be generated, which is the SHG effect. The SHG is a special sum frequency phenomenon. When strong light interacts with matter, the polarization intensity P of the medium can be expressed as a power function of the light field intensity E.

$$P=\varepsilon_{0}\left[\chi^{1}E+\chi^{2}E^{2}+\chi^{3}E^{3}+...+\chi^{n}E^{n}\right]$$

Among them, n = 1 is a linear effect, n ≥ 2 is a nonlinear effect. Generally speaking, the intensity of nonlinear interaction decreases with the increase in n, so the second-order nonlinear effect is the most commonly observed nonlinear interaction. According to the classical nonlinear optics theory, the second-order nonlinear effect is related to electric dipole (ED) polarization, electric quadrupole (EQ) polarization, magnetic dipole (MD) polarization, and higher-order polarization, among which electric dipole polarization contributes the most. In 2D magnetic materials, SHG can usually be divided into I-type SHG and C-type SHG [61], which can be specifically expressed as:

$$\chi^{2}=\varepsilon_{0}(\chi^{\left(i\right)}+\chi^{\left(c\right)})$$

where $\chi^{2}$ is a tensor describing the nonlinear response of the material to the incident field, the time-invariant (I-type) tensor $\chi^{\left(i\right)}$ and the time-noninvariant (C-type) tensor $\chi^{\left(c\right)}$ represent the crystal structure contribution and the magnetic structure contribution, respectively. The C-type contribution only exists when time reversal symmetry is broken. In centrosymmetric magnetic materials, even if the SHG provided by the crystal structure is zero, the non-centrosymmetric magnetic order destroys the spatial inversion symmetry and time reversal symmetry, so a strong SHG signal can be observed. In asymmetric magnetic materials [65], the SHG contributed by the crystal structure and the magnetic structure can exist at the same time. As shown in Figure 1d, when both SHG exist, that is, the spatial inversion symmetry and the time-reversal symmetry are broken at the same time, due to the interference of $\chi^{\left(i\right)}$ and $\chi^{\left(c\right)}$ under the time-reversal symmetry operation, a specific type of nonlinear magneto-optical effect is generated. It can be expressed as:

$$I(2\omega )\propto ({\left|\chi^{\left(i\right)}\right|}^{2}+{\left|\chi^{\left(c\right)}\right|}^{2}\pm 2\left|\chi^{\left(i\right)}\right|\left|\chi^{\left(c\right)}\right|\cos\varphi )E^{4}\left(\omega \right)$$

Here, φ denotes the phase difference between the I-type and C-type susceptibilities. A time-invariant (I-type) term, sensitive to the spinless crystal lattice, and a time-noninvariant (C-type) term, sensitive to the spin lattice and magnetic structure, contribute to the SHG signal. When the spin direction reverses [66], the SHG contribution from the magnetic structure changes its sign, causing the interference term $2\left|\chi^{\left(i\right)}\right|\left|\chi^{\left(c\right)}\right|\cos\varphi$ to invert and the phase φ to shift by 180°. The SHG contributions from the I-type and C-type terms interfere with each other, resulting in the total SHG signal. Applying the principle of nonlinear optics to the study of 2D magnetic materials helps to reveal the magnetic structure, electronic state, and other properties inside the material, and has potential application value in the fields of spin electronics, magneto-optical storage, etc. Among them, SHG has attracted great attention in the study of nonlinear optical properties of 2D magnetic materials, especially for the unique layered structure and magnetic-related behavior of 2D materials, the intensity, layer number dependence [67,68,69,70], and polarization resolution [71,72,73] characteristics of the SHG signal, which can provide important characterization information for the structural properties of the materials.

## 3. Layer- and Polarization-Dependent SHG Spectrum of 2D Magnetic Materials

### 3.1. Layer Dependency

**Figure 2:**
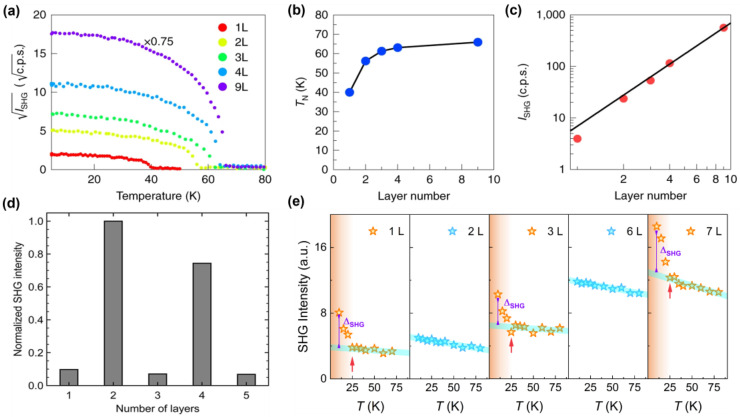
(**a**) Temperature-dependent SHG response of MnPSe_3_ samples with different layers. (**b**) Layer-dependent Néel temperature. (**c**) Layer-dependent second harmonic intensity logarithmic plot [74]. (**d**) SHG response of odd- and even-numbered CrSBr layers [76]. (**e**) Temperature-dependent SHG intensity measured at different thicknesses of CrPS_4_ [65]. panel (**a**–**c**) adapted with permission from Ref. [74], Copyright 2021, Nature nanotechnology; panel (**d**) adapted with permission from Ref. [76], Copyright 2021, Nano letters; panel (**e**) adapted with permission from Ref. [65], Copyright 2024, Advanced Optical Materials.

In the study of 2D magnetic materials, the number of layers is a crucial parameter influencing their intrinsic properties, particularly their nonlinear optical responses. Variations in layer number can lead to significant changes in the SHG intensity. This is because the SHG response intensity is determined by the second-order nonlinear polarization intensity, and is affected by the symmetry of the material. The change in the number of layers will introduce a new symmetry breaking to the original material’s symmetry, which changes the symmetry and, thus, affects the change in SHG response intensity. The dependence of SHG signal intensity on the number of layers is a key variable for understanding the intrinsic characteristics of 2D magnetic materials. Zhuoliang Ni et al. [74] investigated the layer-dependent SHG intensity of MnPSe_3_ as a function of temperature, revealing the Néel temperature and the nearly quadratic dependence of SHG intensity on the number of layers in multilayer MnPSe_3_. As shown in Figure 2a–c, this study extended this investigation to monolayer MnPSe_3_, where similar experiments confirmed that direct imaging of Néel vector switching is feasible in monolayers. This work provides novel insights into domain imaging in bulk antiferromagnetic (AFM) samples. The SHG intensity response exhibits strong dependence on the number of layers, with odd and even layers displaying distinct characteristics due to the material’s structural and magnetic symmetries. Specifically, certain layers exhibit AFM ordering, while adjacent layers display ferromagnetic (FM) ordering. These structural distinctions necessitate consideration of odd- and even-layer contributions in experiments probing the nonlinear optical properties of 2D magnetic materials. Kihong Lee et al. [75] reported that the SHG intensity from even layers, dominated by the ED mechanism, was an order of magnitude higher than that from odd layers. As shown in Figure 2d, this phenomenon arises because the AFM ordering in even layers breaks both spatial inversion symmetry and time-reversal symmetry, enabling ED-driven SHG. In contrast, the FM ordering in odd layers breaks only time-reversal symmetry, leaving spatial inversion symmetry intact and suppressing SHG via the ED mechanism. These findings demonstrate the selective activation of SHG mechanisms based on layer parity. Then, by measuring the temperature dependence of the SHG intensity in the CrSBr bilayer, research proved the disappearance of the central symmetry during the paramagnetic (PM) to AFM phase transition, which is consistent with theoretical knowledge. However, in the subsequent measured PM to FM phase transition, this work also found that the SHG intensity rose rapidly at the critical temperature. This conclusion led them to explore the source of the SHG signal in FM. Therefore, the work theoretically calculated the polarization dependence of the second-order parallel polarization (PP) and cross-polarization (CP) magnetic susceptibility of PM and AFM, and it coincided with the experimentally measured SHG data point diagram of AFM. However, the SHG data point diagram of FM is similar to the SHG data point diagram of AFM, but the SHG intensity of FM does not come from the ED mechanism, but the polarization dependence of the second-order PP and CP magnetic susceptibility of PM and FM under the MD mechanism fits the experimental data points well. De Hou et al. [65] investigated the SHG intensity in CrPS₄ as a function of layer thickness. As shown in Figure 2e, their results show a monotonic increase in SHG intensity with layer thickness. However, the experiment results reveal a nuanced behavior: samples with even layers exhibit a stable I-type SHG response, while odd-layer samples show a temperature-dependent transition. At 25 K (the critical temperature of CrPS_4_), the SHG intensity in odd layers increased sharply, attributed to the phase transition-induced transfer of C-type SHG responses from the uncancelled FM-ordered layers. In contrast, C-type SHG responses in even layers were fully suppressed due to complete AFM ordering. These phenomena underscore the intricate interplay between magnetic ordering, symmetry breaking, and SHG mechanisms in 2D magnetic materials. The observations highlight the importance of considering layer parity and phase transitions in understanding and tailoring the nonlinear optical properties of these systems.

In addition, by compiling and comparing the second-order nonlinear polarization susceptibility ($\chi^{2}$) of different two-dimensional magnetic materials, as shown in Table 1, a clearer understanding can be gained. The differences in $\chi^{2}$ across various materials are visually apparent and reflect not only the thickness, substrate choice, and excitation wavelength but also the unique magnetic and structural properties of each material. For two-dimensional magnetic materials, the SHG intensity is typically closely related to their magnetic properties, particularly for materials that break inversion symmetry. For instance, CrI₃ exhibits a significant $\chi^{2}$ value, indicating that magnetic materials with broken inversion symmetry can generate stronger SHG responses. Its nonlinear response strength is comparable to that of two-dimensional non-magnetic sulfide materials. The differences in the second-order nonlinear response strength of these materials provide strong support for further understanding their potential in practical applications such as photoelectric detection and nonlinear optical devices.

### 3.2. Polarization Resolution

**Figure 3:**
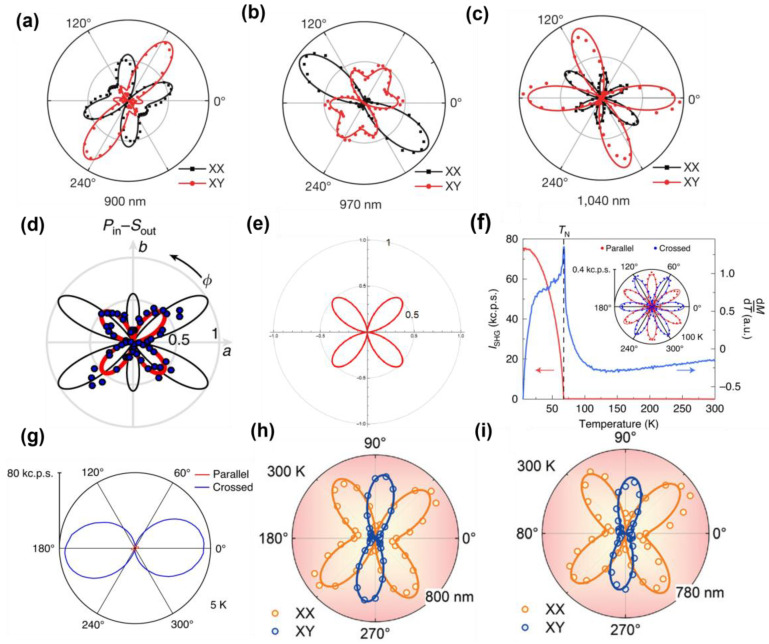
(**a**–**c**) Azimuthal dual-frequency polarization dependence of CrI_3_ irradiated with 900 nm, 970 nm, and 1040 nm lasers at 0 T [17]. (**d**) Comparison of SHG angle resolution experimental data under P_in_-S_out_ polarization (blue dots) to the two predicted types [93]. (**e**) SBHW fit under P_in_-S_out_ polarization shows [94]. (**f**) SHG temperature response diagram of 100 nm MnPSe_3_. (**g**) Polarization-resolved SHG spectrum below T_N_, the node direction in the cross-polarization mode also shows that it is close to the Néel vector direction [74]. (**h**,**i**) SHG anisotropy spectra of CrPS_4_ at 800 nm and 780 nm [65]. panel (**a**–**c**) adapted with permission from Ref. [17], Copyright 2019, Nature; panel (**d**) adapted with permission from Ref. [93], Copyright 2018, Nature communications; panel (**e**) adapted with permission from Ref. [94], Copyright 2023, Journal of the Optical Society of America B. panel (**f**,**g**) adapted with permission from Ref. [74], Copyright 2021, Nature nanotechnology. (**h**,**i**) adapted with permission from Ref. [65], Copyright 2024,Advanced Optical Materials.

The polarization-resolved SHG signals are closely tied to the symmetry properties of the material and have proven to be invaluable in revealing the underlying crystal symmetry and magnetic ordering in 2D materials [92]. For instance, Zeyuan Sun et al. [17] utilized polarization-resolved SHG measurements to uncover the monoclinic stacking order and layered antiferromagnetism in bilayer CrI_3_; they used C-type SHG signal to detect the monoclinic symmetry structure in the bilayer CrI_3_ AFM state., As shown in Figure 3a–c, they showed polarization-resolved images of a double-layer CrI_3_ at three different excitation wavelengths. Apparently, due to the combined spin and inversion symmetry of the material itself, the polarization images at different wavelengths are also different, demonstrating how symmetry-breaking phenomena manifest in SHG signals; this also provides experimental evidence for the monoclinic structure at low temperatures, which is consistent with the prediction. Such measurements provide critical insights into the magnetic and structural properties of the material. Interestingly, polarization studies can sometimes yield unexpected results, leading to novel conclusions about the underlying physics. For materials with layered structures, the angular polarized SHG can still provide a better platform to study the material structure. Given the similarities between perovskite-layered structures and 2D magnetic materials such as CrI_3_ and CrSBr, we have included a discussion and comparison of angle-resolved SHG in perovskites and 2D magnetic materials in our review. Frohna et al. studied the crystal structure of tetragonal MAPbI_3_ as shown in Figure 3d,e and compared experimental SHG data with theoretical predictions, finding that there is no static volume Rashba effect in this type of perovskite [93]. The Rashba effect, arising from strong spin-orbit coupling (SOC) in materials with broken inversion symmetry, leads to a spin–momentum coupling. Based on Frohna’s work, Hardhienata et al. applied the simplified bond-hyperpolarizability model (SBHM) to study the Rashba effect in MAPbI_3_, confirming the absence of static volume Rashba effect, which aligns with Frohna’s conclusions. They further used SBHW fitting to predict the Rashba effect in perovskites with different structures, highlighting symmetry breaking caused by MA ions. Similarly, Sukanya Ghosh et al. studied the Rashba effect in CrI_3_ and found that the interlayer Rashba effect causes spin polarization tilts, leading to energy loss. This indicates that similar Rashba effects occur in both perovskite-layered and 2D magnetic materials, with a stronger effect observed in 2D materials, especially when reduced to a single layer. In summary, both perovskite-layered and 2D magnetic materials exhibit SOC effects driven by symmetry breaking, with observable similarities in the Rashba effect. Zhuoliang Ni et al. [74] investigated the SHG polarization patterns of MnPSe_3_ in collinear and cross-polarization configurations. As shown in Figure 3f,g, while the material’s triple rotational symmetry was expected to yield a six-state clock ground state, the temperature-dependent SHG intensity measurements suggested otherwise. The results indicate that, under strain, the phase transition in the XY model of MnPSe_3_ follows the Ising universality class rather than the XY class. This finding highlights the role of strain-induced symmetry breaking and its impact on the physical properties of 2D materials. Not only that, this study also shows the temperature dependence of the two domains in the same MnPS_3_ crystal. The experiment proves that above the T_c_ temperature, the SHG information is not affected by temperature, and the generated SHG signal only comes from the EQ property of the lattice itself. As the temperature decreases, in the temperature range of 200 K to 78 K, both SHG signals have a slight increase; especially after reaching the critical temperature T_c_, the two SHG signals produce different trends, which is precisely due to the effect of the ED SHG signal.

The SHG intensity of 2D magnetic materials also varies with polarization angles, reflecting the inherent anisotropy of the materials. This relationship between the polarization angle and SHG intensity is a direct manifestation of the material’s structural anisotropy. For instance, De Hou et al. [65] demonstrated that exfoliated CrPS_4_ crystals exhibit a characteristic angle of 67.5° along the Cr diagonal direction. As shown in Figure 3h,i, from a microscopic perspective, this corresponds to the in-plane anisotropy of the CrPS_4_ crystal. Further studies of CrPS_4_ at different wavelengths reveal a direct correlation between the SHG intensity and its anisotropic properties. These findings establish a quantitative link between SHG responses and in-plane anisotropy, offering a powerful tool for characterizing and understanding the optical and structural features of 2D materials. Overall, polarization-resolved SHG experiments provide a rich platform for probing the symmetry, anisotropy, and phase transitions of 2D materials, enabling deeper insights into their magnetic and structural properties while also uncovering the influence of external factors such as strain and temperature.

However, in the process of preparing two-dimensional materials, a large number of samples will inevitably produce structural defects. The manifestation of structural defects in two-dimensional materials generally includes hole defects [95], electron defects [96], grain boundaries [97], etc. The lattice distortion caused by structural defects may cause the anisotropy of the material to change. Generally speaking, structural defects will greatly reduce the properties of the material itself, but it is necessary to consider whether certain specific structural defects can make the material more suitable for promoting various research progress. William Murray et al. studied the effect of structural defects on SHG signals in two-dimensional materials [98]. By irradiating WS_2_ monolayers with focused ion beams to obtain WS_2_ monolayers with structural defects, and comparing them with a simple quadratic relationship, it was found that the SHG signal generated by the irradiated samples decayed much faster than the quadratic relationship used, which caused the magnetic susceptibility of the samples to decrease. However, despite this, the SHG intensity after irradiation only decreased by one order of magnitude relative to before irradiation, and the irradiated samples still had a high SHG signal. Additionally, Chuansheng Xia et al. successfully used SHG imaging to visualize hidden defects in GaN/AlN microdisks by observing the weakening and darkening of the SHG image [99]. This represents one of the most practical applications of SHG technology for defect detection. Moreover, there have been few studies on the impact of defects on SHG in 2D magnetic materials. We believe that with advancements in technology and research, as our understanding of the intrinsic properties of 2D magnetic materials becomes more refined, the influence of defects on SHG will gradually be revealed.

## 4. Magnetic Structure Detection

A significant challenge in the study of 2D magnetic materials lies in the detection of long-range magnetic ordering and magnetic fluctuations. Traditional techniques such as SQUID, vibrating sample magnetometers (VSMs), and neutron scattering have been widely employed to characterize magnetic properties [100,101,102]. These methods offer notable advantages, including high sensitivity and high-resolution measurements of magnetic ordering. However, their limitations become increasingly evident when applied to thin layers and low-dimensional materials. For instance, SQUID and VSMs require relatively large sample volumes and may lack the sensitivity needed to detect weak signals, which are often inherent in low-dimensional materials. Similarly, while neutron scattering and VSMs are highly effective for studying magnetic sequences, their stringent requirements for sample size and uniformity pose challenges for the investigation of 2D materials. These limitations have driven the search for alternative techniques capable of addressing the unique demands of 2D magnetic systems. Optical methods have emerged as promising probes for studying magnetic ordering in 2D materials. Optical techniques provide several unique advantages, such as high spatial resolution, non-destructive measurements, and real-time monitoring capabilities. Among these, SHG has proven particularly valuable. As an optical nonlinear process, SHG is highly sensitive to symmetry breaking and can detect changes in magnetic structures and dynamics in real time without damaging the sample. These features make SHG a powerful tool for investigating the magnetic properties of 2D materials.

In this review, we focus on the application of SHG in the exploration of novel magnetic materials. Specifically, in Section 4 we discuss the high-resolution detection of magnetic ordering, the imaging of magnetic domain structures, and the real-time monitoring of dynamic magnetic processes using SHG. This approach not only overcomes the limitations of conventional methods but also provides deeper insights into the intricate magnetic behaviors of 2D systems, paving the way for advancements in the study and application of 2D magnetic materials.

### 4.1. Detection of Magnetic Sequence

In the study of ferromagnetic materials, a variety of experimental techniques have been widely used, including magneto-optical Kerr effect [103], Raman spectroscopy [104], and tunneling magnetoresistance [105]. These methods can effectively detect the magnetic characteristics, magnetic domain structure and dynamic changes of ferromagnetic materials, and provide rich data and theoretical support. However, in contrast, the research of single-layer antiferromagnetic materials is still in its infancy. There are still many challenges in the detection of long-range magnetic sequences, internal interactions, and related physical phenomena of antiferromagnetic materials.

In this context, the SHG technology shows great potential due to its sensitivity to the nonlinear optical response of magnetic materials, especially in the study of the magnetic characteristics and dynamic behavior of antiferromagnetic materials. The SHG technology is capable of detecting very weak magnetic signals with extremely high sensitivity, especially for detecting complex magnetic structures in antiferromagnetic materials [17,106].

**Figure 4:**
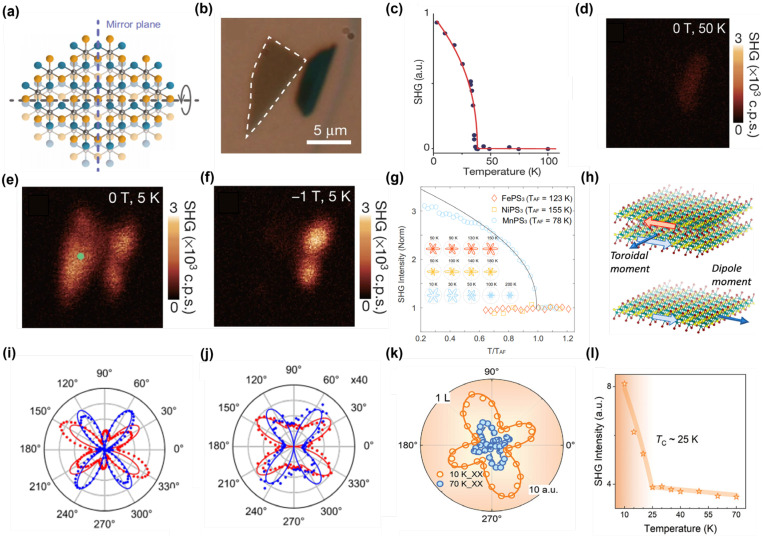
(**a**) Atomic structure of CrI_3_. (**b**) Optical micrograph of a double CrI_3_ layer. (**c**) Non-reciprocal SHG response induced by lamellar antiferromagnetism in a double CrI_3_ layer. (**d**–**f**) Corresponding SHG intensity images when the double layer is in the nonmagnetic, antiferromagnetic, and ferromagnetic states [17]. (**g**) Temperature dependence of SHG intensity of FePS_3_, NiPS_3_, and MnPS_3_ [106]. (**h**) Magnetic symmetry of AFM double layers and FM single layers, with the magnetic toroidal moment T as the AFM order parameter and the net magnetization M as the FM order parameter. (**i**,**j**) Polarization-resolved SHG from CrSBr double and single layers at 65 K (dots) [76]. (**k**) Angle-resolved SHG of a single CrPS_4_ layer, blue is measured at 70 K, orange is measured at 10 K (XX polarization). (**l**) SHG intensity of a single CrPS_4_ layer as a function of temperature [65]. panel (**a**–**f**) adapted with permission from Ref. [17], Copyright 2019, Nature; panel (**g**) adapted with permission from Ref. [106], Copyright 2021, Physical Review Letters; panel (**h**,**i**) adapted with permission from Ref. [76], Copyright 2021, Nano Letters. panel (**k**,**l**) adapted with permission from Ref. [65], Copyright 2024, Advanced Optical Materials.

In 2019, Shiwei Wu and Xiaodong Xu’s [17] group marked the first use of SHG to investigate the magnetic structure and related properties of the 2D magnetic material CrI_3_. In its monolayer form, CrI_3_ exhibits triple rotational symmetry and central symmetry, which prohibits SHG signals under the electric dipole approximation. However, when CrI_3_ forms a bilayer structure in its antiferromagnetic state, the magnetic configuration breaks both temporal and spatial inversion symmetries. This symmetry breaking results in a remarkably strong nonreciprocal second harmonic response. As illustrated in Figure 4a–f, the response coefficient of this nonreciprocal SHG signal is three orders of magnitude higher than that of previously known magnetostrictive SHG signals, such as those observed in chromium oxide (Cr_2_O_3_) [107]. Furthermore, it exceeds by more than ten orders of magnitude the SHG signals generated by conventional ferromagnetic interfaces. Notably, this SHG signal is highly sensitive to changes in the magnetic state of bilayer CrI_3_. When the temperature rises above the antiferromagnetic-to-ferromagnetic phase transition temperature, or when an out-of-plane magnetic field is applied to convert the bilayer CrI_3_ into a ferromagnetic state, the symmetry of the magnetic structure is altered, leading to the disappearance of the SHG signal. This discovery highlights the exceptional capability of SHG in detecting magnetic phase transitions in low-dimensional magnetic systems. The sensitivity and precision of SHG make it a powerful tool for exploring the unique magnetic properties and phase dynamics of 2D materials, paving the way for advancements in the field of low-dimensional magnetism and spintronics [11,12,108]. In addition, two-dimensional antiferromagnetic materials such as MnPS_3_ and FePS_3_ exhibit excellent optoelectronic responses, making them ideal candidates for highly sensitive optoelectronic detection, with a spectral range extending from ultraviolet to near-infrared [109,110]. In the field of nonlinear optics, in 2020, Chu et al. demonstrated that the long-range linear magnetoelectric Néel order in MnPS_3_ can persist down to a thickness of at least 5.3 nm, based on SHG with rotational anisotropy [106]. Despite MnPS_3_, FePS_3_, and NiPS_3_ belonging to a centrosymmetric crystal point group, weak but discernible SHG rotational anisotropy signals have been observed in these materials, even above their Néel transition temperature (T_AF_) (see Figure 4g). This phenomenon may arise from surface electric dipole SHG or higher-order SHG processes, such as EQ SHG, which are generally allowed in centrosymmetric materials. Unlike the zigzag magnetic order in FePS_3_ and NiPS_3_, the Néel-type antiferromagnetic order in MnPS_3_ breaks both time-reversal and space-inversion symmetry below the transition temperature, leading to a significant increase in its second-harmonic signal. By extracting polarization-resolved second-harmonic signals, the magnetic contribution can be distinguished from signals arising solely from lattice inversion symmetry breaking, allowing for a clear observation of the long-range antiferromagnetic order in multilayer MnPS_3_.

To further investigate magnetic ordering in 2D materials, researchers have leveraged the proven capability of SHG to detect antiferromagnetic order and extended its application to studying monolayer ferromagnetic states. However, in high-symmetry lattice structures, the SHG effect dominated by the ED mechanism is insufficient for detecting monolayer ferromagnetism. This limitation arises because inversion symmetry is preserved during magnetic phase transitions in these systems, suppressing ED contributions to the SHG signal. To overcome this challenge, higher-order nonlinear response mechanisms, particularly the MD effect [111], have been introduced as an alternative. The MD mechanism opens new avenues for detecting ferromagnetic order in monolayers. In 2021, Kihong Li et al. [76] used SHG to explore the magnetic order and symmetry characteristics of the 2D material CrSBr. CrSBr, belonging to the orthorhombic crystal system, does not support SHG dominated by the ED mechanism for ferromagnetic monolayers due to its symmetry constraints. Nevertheless, the SHG signal was significantly enhanced below the critical temperature, indicating a transition from a paramagnetic state to a ferromagnetic state. By detecting high-order MD contributions, the researchers identified the ferromagnetic transition temperature of monolayer CrSBr as approximately 146 K. Interestingly, the study also revealed that multilayer CrSBr exhibits antiferromagnetic order. Unlike most other 2D magnetic materials, the Néel temperature of CrSBr increases as the number of layers decreases. This unusual behavior suggests the presence of a unique intermediate magnetic phase, denoted as iFM (intermediate ferromagnetic), between the paramagnetic and antiferromagnetic states. In this phase, the layers exhibit ferromagnetic coupling, while maintaining paramagnetic behavior within individual layers. Using SHG techniques, the researchers further characterized the magnetic dipole moment and magnetic toroidal moment as order parameters for ferromagnetic monolayers and antiferromagnetic bilayers, respectively. As illustrated in Figure 4h,i, this approach provided new insights into intralayer magnetic order and interlayer magnetic coupling in CrSBr. This study not only deepens the understanding of magnetic ordering and phase transition mechanisms in 2D magnets but also underscores the versatility and potential of SHG technology in probing complex magnetic systems. By leveraging higher-order nonlinear optical effects, SHG enables the detection of magnetic states that are inaccessible through conventional ED-dominated processes, paving the way for advancements in the study of 2D magnetic materials.

In recent years, significant progress has been made in the detection of magnetic order in 2D magnetic materials. However, research has predominantly focused on the detection of antiferromagnetic states via SHG dominated by the ED mechanism and the study of ferromagnetic monolayers through SHG influenced by higher-order mechanisms such as MD and EQ. Notably, the detection of ferromagnetic order through SHG under the ED approximation has remained unreported—until 2024 [65], when Hou et al. made a groundbreaking discovery. Their in-depth study of the 2D antiferromagnetic material CrPS4 revealed, for the first time, the presence of a C-type SHG effect induced by ferromagnetic order under the ED mechanism in a 2D magnetic system. This pivotal finding has infused new energy into the field of nonlinear optics. The study demonstrated that C-type SHG signals were absent in bulk CrPS4 and even-numbered layer samples. However, in odd-numbered layers of CrPS4, a strong C-type SHG effect was detected, induced by monolayer ferromagnetic order. As illustrated in Figure 4k,l, this phenomenon is attributed to the simultaneous breaking of spatial inversion symmetry and time-reversal symmetry within the material, enabling an SHG response under the ED mechanism. Additionally, the researchers found that the intensity of the C-type SHG signal induced by ferromagnetic order is comparable to the I-type SHG signal associated with symmetry breaking of the crystal structure. This observation is exceedingly rare among known magnetic materials and suggests the existence of a novel magnetic SHG (MSHG) mechanism.

This discovery provides a transformative perspective on the nonlinear optical responses of 2D magnetic systems. By uncovering the capability of the ED mechanism to detect ferromagnetic order, this work broadens the scope of SHG applications and offers new opportunities for studying magnetic phenomena in low-dimensional materials. The findings not only enhance the understanding of SHG mechanisms in magnetic systems but also pave the way for future innovations in nonlinear optics and magnetism.

### 4.2. Detection of Magnetic Domain Structure and Magnetodynamic Evolution

**Figure 5:**
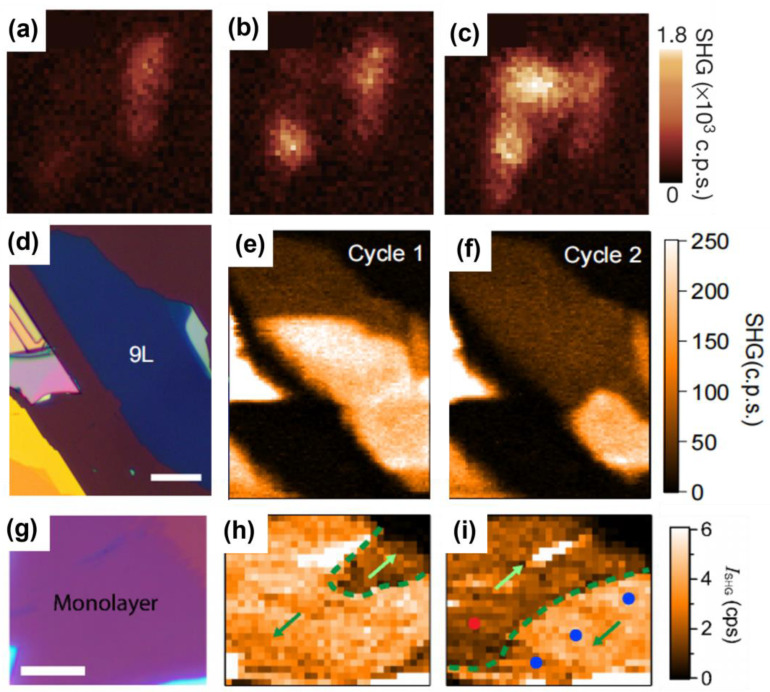
(**a**–**c**) Magnetic transition process of the antiferromagnetic state of bilayer Crl_3_ under magnetic fields of −0.63 T, −0.58 T, and −0.52 T [17]. (**d**) Optical image of 9 L MnPS_3_ sample. Scale bar: 20 µm. (**e**,**f**) SHG intensity mapping measured at 5 K [112]. (**g**) Optical image of 1 L MnPSe_3_ sample. Scale bar: 10 µm. (**h**,**i**) SHG intensity mapping measured at 5 K after different cycles. The dark and bright areas represent two different domains, where the Néel vector switches 180° [74]. panel (**a**–**c**) adapted with permission from Ref. [17], Copyright 2019, Nature; panel (**d**–**f**) adapted with permission from Ref. [112], Copyright 2021, Physical Review Letters; panel (**g**–**i**) adapted with permission from Ref. [74], Copyright 2021, Nature nanotechnology.

SHG also enables researchers to visualize magnetic domain distributions, domain wall structures, and their dynamic evolution with high sensitivity and spatial resolution. In 2019 [17], Zeyuan Sun et al. used SHG technology to study the dynamics of magnetic domains near the magnetic transition, and SHG microscopy to image antiferromagnetic domains in the bilayer CrI_3_. As shown in the Figure 5a–c, the experiments show that the spins of the bilayer CrI_3_ are perfectly aligned at a magnetic field of −0.63 T. When the magnetic field increases to −0.58 T, an SHG signal appears in the lower layer of CrI_3_, indicating the transition of the spin from the alignment state to the interlayer antiferromagnetic state. At a further increase of −0.52 T, the upper layer of CrI_3_ also produces an SHG signal, indicating that more domains switch from the aligned state to the multilayer antiferromagnetic state. The high sensitivity of the SHG makes it possible to monitor the evolution of domain dynamics in real time. In 2D antiferromagnetic materials, the difference in the direction of the magnetic moment will lead to the change of the SHG signal, so that the magnetic domains in different regions can be effectively identified. In particular, the two-state Ising order characteristics and the displacement of the domain wall between the antiferromagnetic regions are clearly observed by SHG measurements at the single-layer limit. In 2021 [112], another study on MnPS_3_ further showed that the Néel-type antiferromagnetic order can be extended to the bilayer structure, and realized the direct imaging of antiferromagnetic domains in 180° reversed phase and the observation of thermally induced domain switching. As shown in the Figure 5d–f, in the same year [74], Zhuoliang Ni et al. successfully imaged the Néel vector switching behavior in the monolayer antiferromagnetic material MnPSe_3_ by SHG technique. In this study, they observed that the SHG signal collapsed into two independent curves during thermal cycling with the help of strain-regulated Ising-type order parameters. As shown in the Figure 5g–i, this phenomenon indicates that the Ising-type Néel vector in the material is switched under the time-inversion operation, and the spatial distribution characteristics of the Néel vector between samples are revealed by SHG imaging. Through these experiments, SHG not only proved its detection accuracy at the single-layer and double-layer limits, but also provided a key experimental method for understanding the spin dynamics and topological characteristics of antiferromagnetic materials.

In addition to experimental research, there has also been progress in theoretical foundations. In order to further clearly understand the relationship between magnetism and SHG effect, Ruichun Xiao and Hui Li [113] recently proposed a new classification method for the second harmonic of magnetically ordered materials. Compared with the traditional classification of second harmonic effect into I-type and C-type, the authors proposed a classification method using isomorphism groups [114,115], which effectively simplified the symmetry analysis process of the SHG effect tensor characteristics under the magnetic point group, and can more clearly reveal the relationship between magnetic ordered structure and SHG. At the same time, a dictionary of SHG effect tensors and a database of magnetically ordered materials were established to provide theoretical support for further detection of magnetic ordered structures.

## 5. SHG Combined with Other Detection Techniques

The rapid development of 2D magnetic materials has attracted widespread attention for their unique magnetic and optical properties. As a sensitive nonlinear optical detection technology, SHG has become an important tool for studying 2D magnetic materials due to its sensitivity to symmetry and magnetic order. However, relying solely on SHG technology may not fully reveal the magnetic characteristics of the material. Therefore, the combination of SHG with other detection techniques, such as MOKE [8,26], Raman spectroscopy [104,116], MCD [58], angle-resolved photoemission spectroscopy (ARPES), etc., provides a more comprehensive perspective for studying 2D magnetic materials.

**Figure 6:**
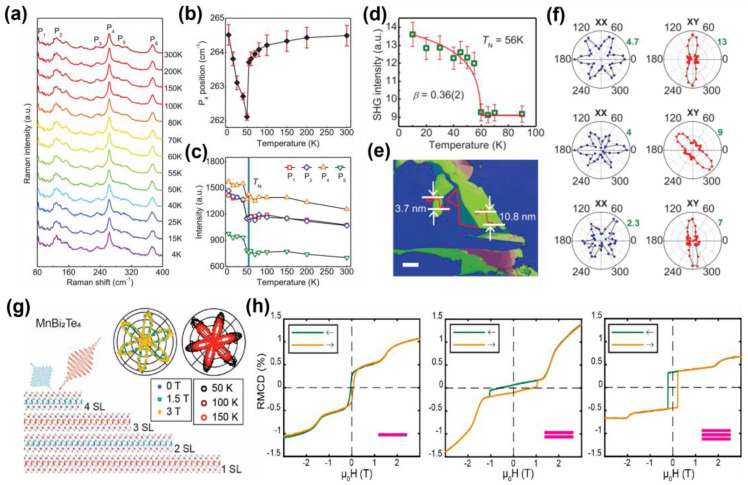
(**a**) Temperature-dependent Raman spectra of bulk VPS_3_. (**b**) Peak position of P_4_ Raman peak at different temperatures from 4 to 300 K. (**c**) Changes in polarized Raman intensity of P_1_, P_2_, P_4,_ and P_6_ with temperature. (**d**) Curves of XY SHG intensity with temperature. The solid red curve is the best fit of the function |1−(T/T_N_)|^2*β*^ (2*β* is the critical exponent). (**e**) Optical image of VPS_3_ exfoliated on SiO_2_/Si substrate. Scale bar: 20 μm. (**f**) XX and XY SHG patterns of 80, 10.8, and 3.7 nm samples at 10 K. (**g**) SHG of MnBi_2_Te_4_ related to layer number, magnetic field, and temperature [120]. (**h**) Reflection magnetic circular dichroism scans of 1 SL, 2 SL, and 3 SL flakes of MnBi_2_Te_4_ measured at 4.5 K [121]. panel (**a**–**f**) adapted with permission from Ref. [120], Copyright 2023, Advanced Materials; panel (**g**,**h**) adapted with permission from Ref. [121], Copyright 2022, Nano letters.

The magneto-optical Kerr effect is an optical detection technique widely used in the study of magnetic materials. By measuring the change in polarization after the reflection of polarized light, the magnetization intensity and direction of the sample can be characterized. When combined with SHG, the two complement each other in detecting magnetic information in different dimensions. In CrI_3_, SHG is used to detect the inversion symmetry breaking within the layer, revealing the transition of its interlayer magnetism from FM to AFM [21]. MOKE provides dynamic information on the change in interlayer magnetic coupling with the external field. Raman spectroscopy is a technique based on the interaction between light and the vibration mode of matter, and is often used to detect the lattice vibration, electron–phonon coupling, and magnetic vibration mode of materials [117]. The combination of SHG and Raman spectroscopy provides new possibilities for the study of the structure and magnetic interaction of 2D magnetic materials. Past studies have shown that Raman spectroscopy can be successfully used to study the Ising-type antiferromagnet FePS_3_. By monitoring a series of new Raman modes that appear due to unit cell doubling during antiferromagnetic ordering, the Ising-type magnetic order of FePS_3_ is retained to the monolayer limit [118]. Kangwon Kim et al. [119] measured the Raman signature of antiferromagnetic ordering in NiPS_3_ and found that the ordering persists up to two layers and is significantly suppressed in the monolayer limit. Chaocheng Liu et al. [120] demonstrated the Néel -type antiferromagnetic order of the out-of-plane spin orientation of VPS_3_. As shown in Figure 6a–f, through temperature-dependent spin–phonon coupling and SHG, Raman spectroscopy analysis shows that the phase transition temperature of VPS_3_ significantly affects its optical properties. Angle-resolved SHG demonstrates the magnetic anisotropy of VPS_3_ and that the Néel vector can persist up to 3.7 nm.

MCD spectroscopy is an optically sensitive probe of surface magnetization of 2D materials. MCD can monitor the magnetic state conversion behavior under the action of external fields (such as temperature and magnetic field) in real time. Combined with the optical technology of SHG, the ability to characterize the spin structure and dynamic behavior of magnetic materials can be further enhanced. In MnBi_2_Te_4_ [121] materials, MCD is employed to probe surface magnetic states and confirm intralayer antiferromagnetic coupling, as well as magnetic phase transitions controlled by external fields. In parallel, SHG has been utilized to study the coupling between crystal symmetry and magnetic structure. Remarkably, as shown in Figure 6g,h, studies have revealed that the SHG signal in MnBi_2_Te_4_ is unexpectedly independent of magnetic state, temperature, and layer number. This suggests that the surface SHG response is the dominant contributor to the overall SHG intensity and is closely aligned with the symmetry of the material. These observations establish a foundational understanding of the nonlinear optical properties of MnBi_2_Te_4_ and highlight its potential in nonlinear optics research. ARPES has proven to be a powerful tool for studying the electronic band structure and spin splitting in materials [122]. When combined with SHG, ARPES can uncover the influence of spin–orbit coupling on magnetic ordering in 2D magnetic systems. ARPES provides detailed insights into the band structure and electronic states, while SHG reveals the nonlinear optical response of the material, creating a complementary approach to studying the interplay between electronic and magnetic properties. Transmission electron microscopy [123] (TEM) offers nanoscale structural information, and its integration with SHG facilitates the correlation between structural and magnetic order properties. In the case of CrPS_4_, researchers used TEM to observe the atomic arrangement in its layered structure [65]. By combining this with SHG data, they analyzed how spatial inversion symmetry breaking influences magnetic SHG. The results demonstrate that local lattice defects significantly affect both the intensity and directionality of the SHG signal. This further verifies the intrinsic connection between lattice symmetry and magnetism in CrPS_4_. These studies underscore the synergistic potential of combining advanced techniques such as SHG, ARPES, and TEM to investigate the intricate relationships between structure, electronic properties, and magnetism in 2D magnetic materials. This multidisciplinary approach offers deeper insights into the fundamental physics of these materials and paves the way for future innovations in spintronics and nonlinear optics.

We have compared the sensitivity, advantages, and limitations of various techniques for probing 2D magnetic materials, as summarized in the Table 2. Sensitivity is evaluated based on the detection limit of the signal, typically quantified by the material thickness, such as a single layer or nanoscale dimension. The combination of SHG and other detection techniques provides a more comprehensive toolbox for the study of 2D magnetic materials. The sensitivity of SHG to symmetry and magnetic state detection complements the high spatial resolution or direct magnetic information characterization offered by other techniques. The joint application of multiple techniques not only enhances the understanding of 2D magnetic materials but also provides a solid theoretical foundation and technical support for the development of new optoelectronic devices. In the future, with further advancements and integration of detection technologies, the potential applications of SHG in the study of 2D materials will become even broader.

Although SHG detection technology has been widely applied in the study of two-dimensional magnetic materials, integrating SHG into practical devices based on these materials is still an emerging research field. Currently, while there have been no direct reports of SHG-based devices, SHG has been successfully used to probe the magnetism, symmetry breaking, and dynamic responses of two-dimensional materials, providing valuable insights for the development of photonic and spintronic devices [17,65]. As our understanding of two-dimensional magnetic materials continues to advance, SHG-based devices are expected to play an increasingly important role in future applications.

## 6. Summary and Outlook

In this review, we have summarized the principles of SHG and the magnetic properties of 2D magnetic materials, highlighting its role in material characterization, magnetic order detection, imaging of magnetic domain structures, and studying magneto-dynamic evolution. Additionally, the integration of SHG with other characterization techniques has been explored, showcasing its potential to advance understanding in the field of 2D magnetism. SHG has proven to be an essential tool for investigating symmetry breaking and magnetic phase transitions, while also offering insights into surface effects and nonlinear optical properties. These advantages position SHG as a valuable method for exploring novel quantum phenomena in low-dimensional systems. Despite its significant contributions, SHG faces several challenges in the study of 2D magnetic materials. A major limitation is the weak signal intensity observed in many 2D materials, particularly in monolayer or sub-nanometer-thick samples, where the nonlinear optical response is further diminished. This issue complicates the extraction and analysis of experimental data, especially when examining subtle magnetic state changes. Achieving sufficient sensitivity in detection systems remains a critical hurdle. Furthermore, the complexity of nonlinear response mechanisms adds another layer of difficulty. SHG signals can arise from ED, MD, and EQ effects, which often overlap and make it challenging to disentangle their individual contributions. Decoupling these mechanisms and quantifying their respective roles requires sophisticated experimental and theoretical approaches. Another significant obstacle lies in conducting SHG experiments under extreme conditions, such as ultra-low temperatures or ultra-high magnetic fields [141,142]. These conditions are essential for studying critical magnetic states, including quantum phase transitions and unconventional magnetic orders. However, the technical difficulties associated with such experiments limit the scope of current research and restrict the detailed exploration of key phenomena.

To address these challenges, future efforts will likely focus on developing materials with enhanced SHG responses and improving experimental methodologies. The discovery of new 2D magnetic materials, particularly those with low symmetry or complex magnetic states, holds promise for advancing the field. Engineering approaches, such as chemical doping and strain modulation, offer potential pathways to optimize the magnetic and nonlinear optical properties of these materials. Additionally, the development of high-sensitivity detection technologies, including optical cavity-enhanced SHG measurement systems, will enable researchers to probe even the weakest nonlinear signals with precision. Combining SHG with complementary characterization techniques, such as ARPES and transmission electron TEM, will provide a more comprehensive understanding of the interplay between structural, magnetic, and optical properties. For instance, the integration of SHG with TEM has already demonstrated its ability to correlate lattice symmetry breaking with magnetic order, offering a clearer picture of how structural defects influence nonlinear optical responses. Meanwhile, theoretical advancements are essential for modeling the complex mechanisms underlying SHG in 2D magnetic materials, providing a robust framework for interpreting experimental data and guiding future investigations.

Looking ahead, the application of SHG in the study of 2D magnetic materials is poised for significant growth. The ongoing discovery of new materials, coupled with advancements in experimental techniques and theoretical models, will expand the scope and depth of SHG research. These developments will not only deepen our understanding of the fundamental physics of 2D magnets but also drive their practical applications in optoelectronics, spintronics, and quantum technologies [141,142]. As a versatile and powerful tool, SHG is set to remain at the forefront of research into the unique properties of 2D magnetic systems.

## Figures and Tables

**Table 1:** Comparison of SHG intensity of different 2D materials.

Material	Thickness	Substrate	*χ*^(2)^ (pm/V)	Excitation Wavelength (nm)	Ref.
Graphene	Monolayer	Si0_2_/Si	90	1300 nm	[77]
WS_2_	Monolayer	Si0_2_/Si	4500 (d_eff_)	832 nm	[78]
MoS_2_	Monolayer	Si0_2_/Si	5000	810 nm	[79]
MoS_2_ (3R)	Monolayer	Si0_2_/Si	405	1200 nm	[70]
ReS_2_	Bilayer	Si0_2_/Si	900	1558 nm	[80]
MoSe_2_	Monolayer	Si0_2_/Si	100	1600 nm	[81]
MoTe_2_ (2H)	Monolayer	Si0_2_/Si	2500	1550 nm	[82]
ε-InSe	20 nm	Si0_2_/Si	13	800 nm	[83]
ε-GaSe	20 nm	Si0_2_/Si	11	800 nm	[83]
GaSe	Monolayer	Si0_2_/Si	700	1600 nm	[84]
GaSe	9 nm (9 L)	Si0_2_/Si	9.3	1560 nm	[85]
GaTe	Monolayer	Si0_2_/Si	1.15	1560 nm	[86]
GaS	Monolayer	quartz	47.98	800 nm	[87]
h-BN	Monolayer	quartz	30	900 nm	[73]
PdSe_2_	Monolayer	Si0_2_/Si	51.7	880 nm	[88]
PbPSe	4.3 nm (6 L)	Si0_2_/Si	64	1300 nm	[89]
NbOI_2_	Monolayer	Si0_2_/Si	190	1050 nm	[90]
Nb_3_SeI_7_	Monolayer	Si0_2_/Si	6.71	1064 nm	[91]
CrI_3_	1.4 nm	Si0_2_/Si	2000	900 nm	[17]
CrPS_4_	Monolayer	Si0_2_/Si	1	800 nm	[65]

**Table 2:** Comparison of sensitivity, advantages, and limitations of various techniques for probing 2D magnetic materials.

Technique	Sensitivity	Advantages	Limitations	Refs.
SHG	High (sub-monolayer sensitive)	Probes symmetry breaking Detects AFM/FM order Sensitive to time-reversal symmetry	Limited to nonlinear effects Challenges with quantification	[17,120,121,124,125]
MOKE	High (monolayer sensitive)	Non-destructive Maps magnetic domains High spatial resolution	Primarily surface-sensitive Limited sensitivity for AFM	[103,126,127,128]
MCD	High (surface-sensitive, ~monolayer)	Sensitive to spin-polarized states Provides information about electronic transitions Non-destructive	Less effective for low symmetry or AFM materials	[129,130,131]
ARPES	High (surface-sensitive, ~monolayer)	Energy resolution down to meV Detecting electronic band structures and spin-polarized states	Requires ultra-high vacuum conditions. High sample preparation requirements Restricted in resolving bulk electronic structure	[132,133,134]
Raman	High (monolayer sensitive)	Strain and defect mapping Sensitive to magnetic phonons	Less direct for magnetic ordering	[43,118,119,135]
Neutron scattering	Low (bulk-sensitive)	Non-destructive Direct magnetic probing	Limited to large sample size High sample quality requirement Expensive setup	[136,137,138]
Magnetometry (SQUID, VSM)	Low (bulk-sensitive)	Measures bulk magnetization	Requires sample in bulk form	[139,140]
